# Decision, Sensation, and Habituation: A Multi-Layer Dynamic Field Model for Inhibition of Return

**DOI:** 10.1371/journal.pone.0033169

**Published:** 2012-03-12

**Authors:** Jorge Ibáñez-Gijón, David M. Jacobs

**Affiliations:** 1 Facultad de Psicología, Universidad Autónoma de Madrid, Madrid, Spain; 2 Facultad de Filosofía, Universidad de Murcia, Murcia, Spain; Baycrest Hospital, Canada

## Abstract

Inhibition of Return (IOR) is one of the most consistent and widely studied effects in experimental psychology. The effect refers to a delayed response to visual stimuli in a cued location after initial priming at that location. This article presents a dynamic field model for IOR. The model describes the evolution of three coupled activation fields. The decision field, inspired by the intermediate layer of the superior colliculus, receives endogenous input and input from a sensory field. The sensory field, inspired by earlier sensory processing, receives exogenous input. Habituation of the sensory field is implemented by a reciprocal coupling with a third field, the habituation field. The model generates IOR because, due to the habituation of the sensory field, the decision field receives a reduced target-induced input in cue-target-compatible situations. The model is consistent with single-unit recordings of neurons of monkeys that perform IOR tasks. Such recordings have revealed that IOR phenomena parallel the activity of neurons in the intermediate layer of the superior colliculus and that neurons in this layer receive reduced input in cue-target-compatible situations. The model is also consistent with behavioral data concerning temporal expectancy effects. In a discussion, the multi-layer dynamic field account of IOR is used to illustrate the broader view that behavior consists of a tuning of the organism to the environment that continuously and concurrently takes place at different spatiotemporal scales.

## Introduction

Inhibition of Return (IOR) is a phenomenon related to spatial orientation behavior. The phenomenon consists of an increase in Response Time (RT) for targets appearing at a peripheral location after spatially informative cues, as compared to targets appearing after uninformative cues. The inhibitory effect is observed if the Cue-Target Onset Asynchrony (CTOA) is longer than a certain task-dependent value. For shorter CTOAs priming occurs: cued targets lead to faster responses than uncued ones. Since Posner and Cohen's foundational work [1], IOR has been investigated with a wide variety of experimental procedures, leading to a substantial body of knowledge about the circumstances under which the effect occurs (see [2]–[4] for reviews). IOR has been related to sensorimotor interactions in the oculomotor system. The aim of our modeling efforts is to precisely formalize a sensorimotor hypothesis. To describe the motivation for the model in more detail we briefly review the evidence that relates IOR to the oculomotor system.

A first line of evidence that relates IOR to the oculomotor system is provided by clinical studies. One of the main neurophysiological structures of the saccadic control system is the superior colliculus. Patients with midbrain degeneration due to progressive supranuclear palsy, who can be assumed to have the superior colliculus affected, show abnormal RTs in IOR tasks [5]. On the contrary, IOR is preserved in patients with hemianopsia, for who the retinotectal system—which includes the superior colliculus—is intact [6]. Relatedly, IOR is observed in studies with newborns, whose vision is predominantly mediated by the retinotectal system [7]. To summarize, these clinical studies indicate that IOR is observed for individuals with an intact superior colliculus, but not for individuals with a damaged superior colliculus, hence providing evidence for the implication of the superior colliculus in IOR.

More direct evidence is provided by single-unit recordings in the superior colliculus of monkeys [8]–[11]. The saccadic behavior of monkeys in IOR tasks is qualitatively similar to the behavior of humans, with early facilitation and late inhibition at cued locations. This behavior is paralleled by the activity of visuomotor neurons in the intermediate layer of the superior colliculus. The effect of the target, measured as the difference between the activity of the neurons before and after the presentation of the target, is consistently depressed in cue-target-compatible stimulations, even at the shortest CTOAs. This depression does not lead to IOR at short CTOAs because the cue increases the pretarget activity. The cue-induced pretarget activity adds to the target-induced increase in activity, overcompensating the depressing effect of the cue and hence resulting in a faster response. IOR is observed at longer CTOAs because the increase in pretarget activity caused by the cue decays more quickly than the depressing effect of the cue.

Neurons in the intermediate layer of the superior colliculus are not less sensitive to electrical stimulation in cue-target-compatible situations [9]. This indicates that the depression of the effect of the target in cue-target-compatible situations is due to the fact that the intermediate layer of the superior colliculus receives less intense stimulation, and thus that factors that contribute to IOR are located earlier in the sensory stream. Fecteau and Munoz analyzed the activity of visual neurons located in the superficial layer of the superior colliculus [11]. The activity of these neurons is indeed depressed in the cue-target-compatible situations, supporting the view that IOR reflects a habituated response in earlier sensory areas.

The above-mentioned neurophysiological studies were used by Dukewich as support for her reconceptualization of IOR [12]. Dukewich's work is based on the concept of orienting response, which is traditionally used to describe the orienting of the sensory organs toward novel events in the environment. Examples of such events are the cues and targets in IOR experiments. It is well known that the strength of the orienting response decreases with the repeated presentation of stimuli. In other words, the orienting response shows habituation. Dukewich's portrayal of IOR is as follows: A spatially informative cue leads to habituation of the orienting response, causing a slower reaction to targets that are presented at the same location as the cue.

In sum, the available evidence indicates that IOR is related to oculomotor interactions in the superior colliculus and to earlier sensory habituation. Our multi-layer model for IOR is inspired by this evidence. In line with the portrayal of Dukewich [12], the model relies on the concept of habituation, and in line with single-unit recordings [8]–[11], the habituation is included in one of the layers of the model (the sensory layer) but not in another layer (the decision layer). More broadly, we developed the model inspired by the view that behavior consists of a multi-scale tuning of the organism to the environment—an issue that is addressed in more detail in the Discussion.

### Modeling

#### Dynamic Field Models and IOR

Sensorimotor accounts of IOR hold that the inhibitory effect emerges from complex interactions in the oculomotor system. Dynamical models provide useful conceptual insights and mathematical tools to study such interactions, because these models allow one to quantitatively and qualitatively inspect the spatiotemporal evolution of the interactions and to propose concrete and falsifiable hypotheses. The specific dynamic modeling approach that we use in this study is referred to as *dynamic field approach* [13].

The starting point of a dynamic field model is a continuous space that is in many cases hypothesized to correspond to a spatially organized map in a specific brain area. Two closely related functions are defined on the space: A first function describes the internal activation of loci in the space, referred to as neurons, and a second function describes the external activation, or firing rate, of the neurons. The goal of the modeling is to describe how these activation functions evolve over time. Factors that contribute to the evolution are: (1) spontaneous decay of the activation, (2) lateral interaction among loci in the continuous space, and (3) input from other brain regions. The input from other brain regions is usually divided in exogenous and endogenous input to indicate their relative proximity to sensory surfaces. A precise description of each of these factors results in a system of integro-differential equations that is solved numerically so as to determine the behavior of the system.

A dynamic field model for IOR that is closely related to ours has recently been reported by Satel and colleagues [14]. The model of these authors consists of a single layer and the model assumes habituation (as observed in [11]) as an *ad hoc* modification of the sensory input. The main contribution of our model beyond the one presented by Satel and colleagues is that our model consists of multiple layers (cf. [15]). Multi-layer models allow one to explain phenomena that arise from the interaction of processes with different time-scales. In our view IOR is such a phenomenon because it arises from the fact that the decay of the cue-induced pretarget activity is quicker than the decay of the cue-induced depression of the effect of the target. An additional contribution of our model is that it includes a dynamic account of habituation.

#### Model Equations

**Figure 1:**
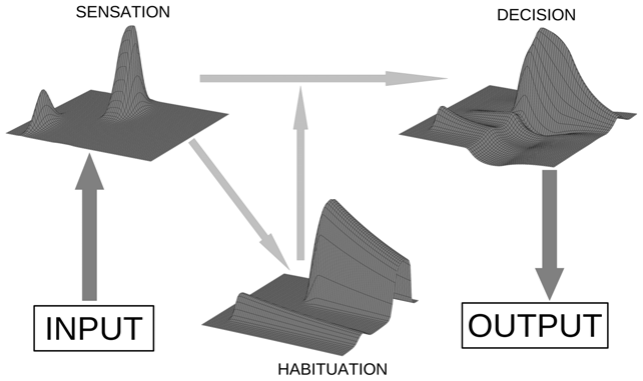
Schematic representation of the structure of the model. Input into the model is received by a sensation field, which is mutually coupled to an activation-dependent habituation field. The decision field receives input from the sensation field and triggers a response upon reaching a threshold. Time and space are represented by left-right and in-depth dimensions, respectively.

Figure 1 presents a schematic diagram of the interactions within our model, using the fields obtained in a sample simulation. We next describe these interactions in more detail. The internal and external activation of the *decision field* are denoted as *D(x,t)* and *a_D_(x,t)*, respectively, with *x* and *t* indicating the spatial and temporal dimensions. The decision field implements accumulating evidence for a motor decision. A decision is triggered when the external activation reaches the threshold of 80% of the maximal activation. The decision field is inspired by the intermediate layer of the superior colliculus and does not suffer habituation; rather, in cue-target-compatible situations the field receives reduced sensory input. The reduced input comes from the second field, the *sensory field*, which is assumed to reflect earlier sensory processes. The internal and external activation of the sensory field are denoted as *S*(*x*,*t*) and *a_S_(x,t)*. The sensory field suffers habituation, meaning that with a sustained activation of the field the same internal activation comes to lead to less and less intense external activation. The habituation of the sensory field is implemented with a third field, the *habituation field*, denoted as *H(x,t)*.

The equations of our model are closely related to the ones in previous dynamic field models [13], [14], [16]. The evolution of *D*(*x*,*t*), *S(x,t)*, and *H(x,t)* is described by:
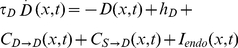
(1)

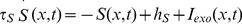
(2)

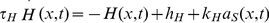
(3)The constants *τ_D_* = .328 s^−1^, *τ_S_* = .048 s^−1^, and *τ_H_* = 1.620 s^−1^ encode the relative timing of the processes: The higher a *τ*, the lower the temporal derivative that it multiplies, and, as a consequence, the slower the process. The constants that we used were chosen so as to optimize the fit with data reported by Posner and Cohen [1].

The first two terms on the right-hand sides of Equations 1 to 3 determine the decay of the fields to their resting levels. The used resting levels were: *h_D_* = −30, *h_S_* = −1, and *h_H_ = 0*. The remaining terms in the equations are described in sequential order, starting with the connectivity terms, 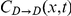 and 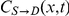, and proceeding with the endogenous and exogenous input terms, *I_endo_*(*x*,*t*) and *I_exo_*(*x*,*t*). However, because the connectivity terms are defined with the external activation functions, we first describe these activation functions and their relation to the habituation.

Remember that *D*(*x*,*t*) and *S(x,t)* represent the internal activation of the fields, or, more precisely, the internal activation of the neurons, or *x*-loci, of the fields. The internal activation is related to the external activation, or spike rate, through the equations:
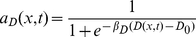
(4)

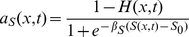
(5)where the constants are β*_D_* = 1.4, β*_S_* = 6, *D_0_* = 0, and *S_0_* = 0. These functions are sigmoids with slopes parameters β*_D_* and β*_S_*. If the slopes are high, then the internal activation is transformed into an approximately bistable system that is either close to 1 or close to 0, to be interpreted as a neuron that either spikes or does not spike.

Equation 5 includes the habituation. With our parameter settings the habituation always remains between 0 and 1. No habituation occurs with *H(x,t) = 0*, in which case the maximum achievable spike rate is 1. The more *H(x,t)* approaches the value of 1, the lower the maximum achievable spike rate. Reciprocally, the habituation *H(x,t)* depends on the activation *a_S_(x,t)*, as defined in Equation 3. The habituation increases when the sensory field is spiking. To implement the behaviorally observed asymmetry in habituation, with a relatively fast build-up and a slow decay, we used *k_H_* = 7. A similar way to implement activation-dependent habituation can be found in [17]. Having defined Equations 4 and 5 we are now in the position to address the connectivity terms.

The term 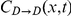 in Equation 1 represents the overall excitation or inhibition received by a neuron in the decision field from other neurons in the decision field. This is referred to as lateral interaction. The lateral interaction is given by a convolution:
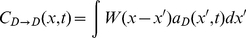
(6)with
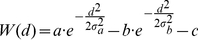
(7)where *a = 11*, *b = 4.5*, *c = 1*, *σ_a_ = 4* and *σ_b_ = 7*. The function *W(d)* encodes the connectivity among neurons in the decision field. Following [16], we used a Mexican hat pattern defined with a double Gaussian function, which depends only on the distance between the neurons. The Mexican hat operator implements excitation for near loci and inhibition for distant loci.

For the influence of the sensory field on the decision field we used a 1-to-1 projection:
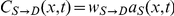
in which 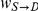 = 95. We now turn to the encoding of the input, again largely following previous work [13], [14], [16].

Stimuli were encoded as Gaussian distributions of excitation. Exogenous stimuli decayed. This decay models the fact that exogenous stimuli affect the sensory field mainly when they appear. The equations of the stimuli, or input, are:
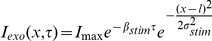


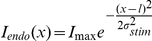
The location *l* corresponds to the center of the input and τ gives the time since the stimulus onset. The used value of the decay parameter was β*_stim_* = .07 s^−1^. Exogenous input was included for cues (*I_max_* = 40; σ*_stim_* = 8) and targets (*I_max_* = 60; σ*_stim_* = 8). The duration of the cues was 50 ms. Targets lasted until the model reached the response threshold. A small endogenous preactivation, constant over time, was included at the fixation point (*I_max_* = 10; σ*_stim_* = 4). The widths of the input distributions are given in units that correspond to the distance between successive neurons in our numerical approximations.

A final endogenous effect included in the model concerns the expectation or foreperiod effect [18]. In typical IOR tasks, cues are either informative or not informative about the spatial properties of the target, but they are always informative about the temporal properties of the target. That is, a target usually appears a certain time-interval after the cue, even though the length of that time interval may vary. This means that the conditional probability that a target appears, given that it has not appeared yet, increases during the interval after the cue in which the target may appear. Experimentally observed RTs parallel this increase in probability: the longer after the cue the target appears, the shorter the RT. To model this expectation effect we included endogenous signals (σ*_stim_* = 8) at each of the possible locations of the target (cf. [14]). The *I_max_* of these signals increased over time:
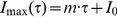
(8)where *m* = 50 *s^−1^* and *I_0_* = 18.

Following previous work [14], perceptual delays were used to represent the time needed for the input to reach the respective fields. For exogenous input the delay was 70 ms and for endogenous input the delay was 120 ms. A motor delay of 80 ms was used to represent the time between a decision (i.e., *a_D_[x,t]* reaching threshold) and the registration of a response.

Dynamic fields can show different types of attractors. A first type of attractor consists of a region with a high level of activation that sustains itself with nearby excitation of the loci in the region, while the activation of the rest of the field is depressed with long-range inhibition. A more trivial attractor occurs when all neurons approximate the resting level. With our parameter settings the self-sustained type of attractors did not occur. This means that the model operated in the input-driven regime: In the absence of input the fields decayed toward their respective resting levels (as can be seen in Figure 1).

## Methods

Parameters were optimized with data estimated from Figure 32.2 of the influential chapter by Posner and Cohen [1]. In the considered experiment, the outlines of three squares appeared at the start of each trial. Cues were implemented by the brightening of the outline of the left or right square. Targets were implemented by a smaller square that appeared in one of the outlines. The task consisted in responding with a manual keystroke to the target. Instructions were to move as fast as possible while fixating the center square. Six CTOAs were used: 0, 50, 100, 200, 300, and 500 ms.

To numerically simulate the behavior of the coupled fields we considered 100 equidistant nodes, or neurons, per dynamic field. We used two exogenous signals: one each for cue and target, centered at Node 25 or Node 75. Catch trials at which the target appeared at Node 50 were not included in the analyses. Endogenous expectation signals were used at each position at which a target could appear in the experiment: Nodes 25, 50, and 75. An additional endogenous signal at Node 50 represented the fixation instruction.

The differential system that defines the model is well behaved with regard to integration techniques (cf. [13]). We therefore used a first-order Euler algorithm for the integration, with a step-size of 5 ms. This is a fast algorithm that requires only the first temporal derivative.

The models were fitted to the experimental data with a deterministic evolutionary algorithm [19] that optimized the differences in the RTs for cued and uncued trials—which is to say that it optimized the predicted IOR. More precisely, the fitness function, *F*, was:
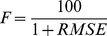
(9)The Root Mean Squared Error (RMSE) in this formula was defined as follows:
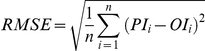
where *n* is the used number of CTOAs (*n*  =  6), the subscript *i* indicates a particular CTOA, *PI_i_* is the inhibition (RT_cued_-RT_uncued_) predicted by the model for a particular CTOA, and *OI_i_* is the experimentally observed inhibition for that CTOA. Hence, the lower the differences between the predicted and observed inhibition, the higher the fitness, with the theoretical maximum of the fitness being 100.

The parameters included in the evolutionary optimization were: *τ_D_* (ranging from .01 to 1 s^−1^), *τ_S_* (ranging from .01 to 1 s^−1^), *τ_H_* (ranging from .01 to 2.5 s^−1^), 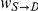 (ranging from 0 to 100), *m* (ranging from 1 to 100), and *I_0_* (ranging from 0 to 20). The delay parameters were taken from [14]. The remaining parameters were set by hand.

The hardware platform used to perform the simulations was an Intel i7-930 processor with 12 GB of RAM running a Debian GNU/Linux 6.0 environment. All simulations and analyses were performed using Python scripting language, Scipy/Numpy for multidimensional array manipulation, and Matplotlib for plotting. Integration of differential equations was implemented in a self-developed Python C library extension. Genetic algorithm code was also self-developed.

## Results

Our main challenge in this research is to explain the basic mechanisms underlying IOR using a multi-scale dynamics paradigm. Simulations were performed to replicate behavioral data, to compare the simulated activation of neurons in the model to measured neurophysiological data, to analyze habituation, and to analyze the expectation effect.

### Simulating Behavioral Data

**Figure 2:**
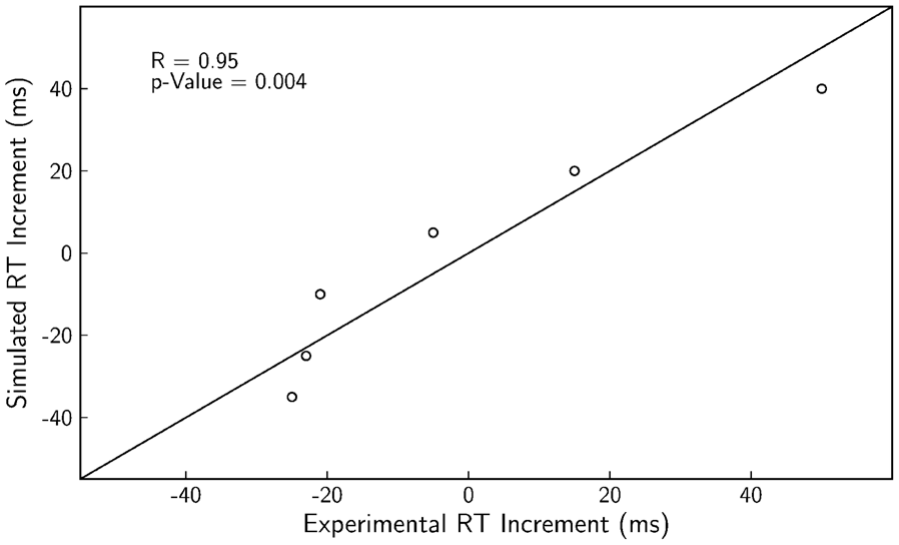
Comparison of simulated and experimental IOR. RT increments for simulated data plotted against RT increments for experimental data. Each dot reflects a single CTOA. Experimental data were estimated from Figure 32.2 in [1]. The figure indicates that the optimization resulted in good fits.

Figure 2 shows simulated RT increments plotted against experimental RT increments (estimated from [1]). The increments reflect RTs for uncued trials subtracted from RTs for cued trials, hence providing a measure of IOR. The dots closely approximate the diagonal, indicating that the simulated data closely approximate the experimental data. The achieved fitness value (Equation 9) is 99.1. The average absolute difference between simulated and experimental RT increments is 8.0 ms (SD = 3.3). Also shown in the figure are the Pearson product-moment correlation between simulated and experimental data (*r* = .95) and the associated significance level (*p* = .004). Model parameters were optimized with the data shown in this figure; all subsequent analyses used the same parameter values.

**Figure 3:**
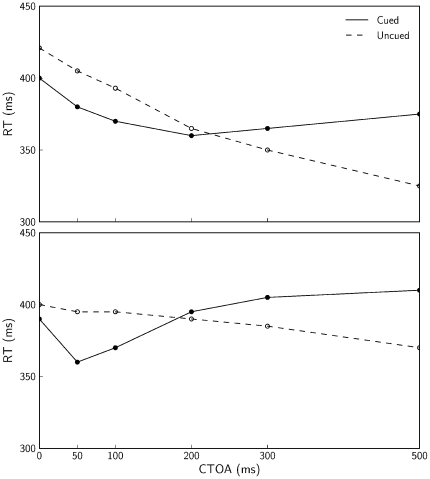
Mean RTs for experimental and simulated data. Mean RTs are shown for different CTOAs. Upper panel shows behavioral data estimated from Figure 32.2 of [1] and lower panel shows results of our simulations. The figure indicates reasonable fits despite the fact that these means were not included in the optimization algorithm.

Figure 3 provides a more detailed comparison of the experimental and simulated data (upper and lower panel, respectively). Presented are the mean RTs for cued target (filled dots) and uncued targets (open dots). The average absolute difference between the experimental and simulated data shown in this figure is 23.3 ms (SD = 14.6) for the cued trials and 23.0 ms (SD = 14.4) for uncued trials. Even though these means per condition were not included in the optimization algorithm, the errors are relatively small, hence indicating that the model reflects the characteristic behavioral features of IOR.

Better fits were observed for optimizations that excluded the condition CTOA = 0, in which the experimental data show more facilitation (21 ms) than the current simulations (10 ms). In the condition CTOA = 0 the cue and target appear at the same moment. The model assumes a linear summation of the inputs of cue and target which, apparently, did not lead to sufficiently pronounced reduction in RT. Our interpretation of this result is as follows: with the target, the external activation of the sensory field already tends to approach its maximum of 1, reducing the possible facilitatory effect of additional activation caused by the cue. We did not try to achieve *ad hoc* improvements of the model in this regard.

### Simulating Neurophysiological Data

**Figure 4:**
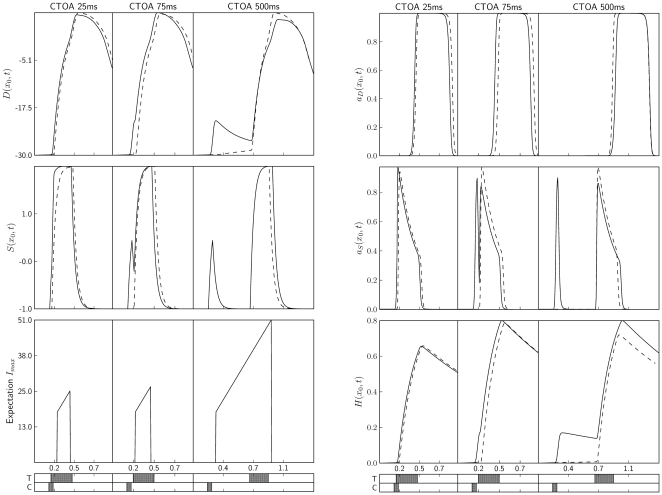
Component-by-component overview of the functioning of the model. Evolution of *D(x_0_,t)*, *S(x_0_,t)*, and *I_max_*-parameter of expectation signal (left panels) and of *a_D_(x_0_,t)*, *a_S_(x_0_,t)*, and *H(x_0_,t)* (right panels), as a function of time. All signals are illustrated for representative CTOAs: 25, 75, and 500 ms. The activation for cued trials is indicated with solid curves and the activation for uncued trials with dashed curves. T = timing of the target; C = timing of the cue. The figure provides intuitions about the functioning of the model, most particularly about the interplay of early facilitation and late inhibition. See text for details.

To compare our model to neural activation measured with single-unit recordings, and to provide an intuition about the functioning of the model, we next consider the activation of simulated neurons at the *x*-locus of the target, referred to as *x_0_*. Figure 4 depicts the temporal evolution of the components of the model under different stimulation conditions. The left part of the figure contains, from top to bottom, the time course of the internal components of the model, *D(x_0_,t)* and *S(x_0_,t)*, and the time course of the *I_max_*-parameter of the expectation signal. The right part of the figure contains the external activation variables, *a_D_(x_0_,t)* and *a_S_(x_0_,t)*, and the habituation, *H(x_0_,t)*. All variables are presented for CTOAs of 25, 75, and 500 ms. Solid curves indicate cued targets and dashed curves indicate uncued targets. The timing of the target (T) and the cue (C), taking into account the delay of these signals, is indicated at the bottom of the figures.

The presented CTOAs where chosen to illustrate the possible summations between cue and target. For CTOA = 25 ms there is temporal coincidence of cue and target, whereas for CTOA = 75 ms there is no temporal coincidence, but there still is summation, for instance in *S(x_0_,t)*, of the cue-induced and target-induced activation. For CTOA = 500 ms, the excitation generated by the cue has dissipated from *S(x_0_,t)* due to the fast temporal dynamics of the sensory field. Behaviorally, the first two CTOA conditions show facilitation for informative cues and the last CTOA condition shows inhibition. Let us consider the observed dynamics for each stimulation condition in more detail.

In the CTOA = 25 ms condition, the coincidence of cue and target produces a higher activation in *S(x_0_,t)* for cued trials than for uncued trials, but this difference is partially mitigated in *a_S_(x_0_,t)*. The difference in stimulation received by *D(x_0_,t)* can be measured as the area between the dashed and solid curves in *a_S_(x_0_,t)*, which is small. Differences in RTs were computed as the differences in which *any* locus in the *a_D_(x,t)* field reached the threshold of .8. These differences, however, are essentially the same as the differences in which the target-aligned curves in the *a_D_(x_0_,t)* plots reach the threshold. The leftmost panel for the *a_D_(x_0_,t)* signal shows that cued trials are faster than the uncued ones, but not much (see also Figure 3).

In the CTOA = 75 ms condition, the temporal succession of the cue and target determines that the difference in the excitation of *a_S_(x_0_,t)* that is projected to *D(x_0_,t)* in the cued and uncued conditions is larger than in the CTOA = 25 ms condition. The curves *S(x_0_,t)* and *a_S_(x_0_,t)* now show an almost independent peak of activation for the cue. The peak in *a_S_(x_0_,t)* raises the activation in *D(x_0_,t)* so that, when the target arrives, *D(x_0_,t)* already has a substantial activation and *a_D_(x_0_,t)* more easily reaches the threshold. This facilitatory effect goes together with an inhibitory effect. The stronger and earlier habituation in the cued condition, which can be observed in *H(x_0_,t)*, leads to a lower target-induced peak in *a_S_(x_0_,t)*, and hence to a smaller target-induced effect in *D(x_0_,t)*. Because in this CTOA condition the facilitatory effect is stronger than the inhibitory effect, the solid curve for *a_D_(x_0_,t)* reaches the threshold sooner than the dashed curve.

In the CTOA = 500 ms condition, the temporal interval between cue and target is so large that *S(x_0_,t)* and *a_S_(x_0_,t)* reach their resting state before target onset. When the target-induced excitation arrives, there is little cue-induced excitation left in *D(x_0_,t)*. The dominant component of the dynamics, therefore, is the slow temporal evolution of *H(x_0_,t)*, giving rise to an inhibitory effect. The magnitude of the inhibitory effect can be observed in the difference in the peaks generated by the target in *a_S_(x_0_,t)*. This difference is propagated to *D(x_0_,t)*, where uncued trials now have a higher target-induced slope, producing a faster RT.

The interplay of the facilitatory and inhibitory effects in the model shows a qualitative resemblance to the activation of visuomotor neurons in the intermediate layer of the superior colliculus. It is illustrative to compare our simulation results to the single-unit recordings of Fecteau and Munoz [11] and to the schematic portrayal of IOR as the product of habituation by Dukewich [12]. In both examples, facilitation at short CTOAs goes together with the summation of target-induced and cue-induced activation (Figure 2c of [12]; blue line in upper right panel of Figure 3 of [11]), and inhibition at longer CTOAs goes together with a reduced target-induced effect and a by then largely decayed cue-induced activation (Figure 2d of [12]; blue lines in lower right panels of Figure 3 of [11]).

One cannot expect more than a qualitative similarity between our simulation results and the neurophysiological recordings because the model was optimized for manual RTs of humans and the recordings concerned saccadic responses of monkeys. One of the notable differences between the simulations and the neurophysiological data is that we observed a cue-induced peak of activation in *D(x_0_,t)*, most clearly in the CTOA = 500 ms condition, but not in *a_D_(x_0_,t)*. The field *a_D_(x,t)*, however, is hypothesized to reflect external activation in the intermediate layer of the superior colliculus, and cue-induced peaks of activation have been measured in this layer [11]. It is possible to make the cue-induced activation more visible in the model component *a_D_(x,t)*, for instance by setting *h_D_* in Equation 1 closer to 0 or by using a smaller value of *β_D_* in Equation 4.

### The Effect of Habituation

The sensorimotor hypothesis affirms that IOR is related to habituation and habituation-induced sensory depression. To inspect the sensory depression in the model, we ran simulations that were identical to the previous ones with the following exceptions: CTOAs were varied from 0 to 4 s in increments of 10 ms, the target lasted 500 ms, and we used *I_exo_* = 60 for cue and target.

**Figure 5:**
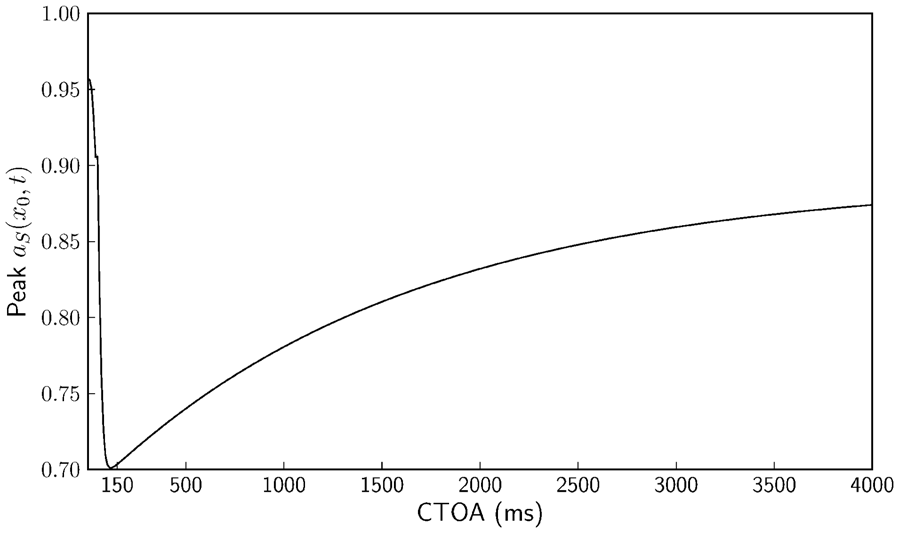
Habituation curve. Peak value of *a_S_(x_0_,t)* in response to a 500-ms target after a 50-ms cue, as a function of CTOA. The figure indicates a qualitative similarity of our dynamically generated sensory depression to experimentally observed sensory depression [11].

Figure 5 shows the maximum height of the target-induced peak in *a_S_(x_0_,t)*, hence illustrating the sensory depression. The curve quickly drops for small CTOAs, reaches a minimum at a CTOA of about 150 ms, and slowly recovers for higher CTOAs. Remember that the habituation in the model is generated dynamically, which is to say that it is fully accounted for by a simple set of equations. Figure 5 may be compared to the left side of Figure 4b of [11] (cf. Figure 1c of [14]). These authors measured the target-induced activity in visual neurons in the superficial layer of the superior colliculus. The simulated curve is similar to the measured one in the sense that it shows a fast initial drop in target-induced activity followed by a gradual increase for longer CTOAs.

Let us note once more that the data of Fecteau and Munoz [11] were measured for monkeys performing a saccadic reaction task. IOR for saccadic responses of monkeys is typically observed only for CTOAs smaller than 1 s, whereas IOR for manual responses of humans may last as long as 6 s. This difference is reflected in the respective habituation curves: Whereas the curve of Fecteau ad Munoz shows a full recovery for CTOAs of about 1 s, our curve shows habituation for CTOAs of up to 4 s.

### The Effect of Temporal Expectation

**Figure 6:**
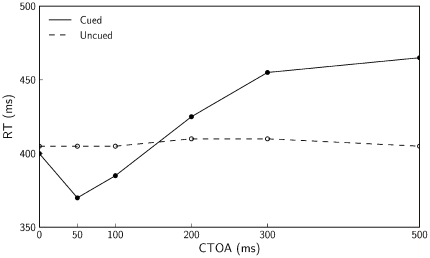
Simulations without temporal expectation. Simulated RTs for different CTOAs obtained with a model without endogenous expectation-related input. The figure indicates that the characteristic RT increments are observed also for a model without expectation input.

Our model emphasizes sensorimotor contributions to IOR. Even so, the model includes endogenous input for the temporal expectation effect (Equation 8; lower panels of left side of Figure 4), which can be interpreted as an influence from higher-order processes [20]. To test the importance of this input we performed simulations without it. The mean RTs obtained with these simulations are presented in Figure 6. The distance between the curves in the figure indicates that the model without expectation input reproduces IOR. Furthermore, whereas the RTs produced with the expectation input show a tendency to decrease with increasing CTOAs, especially for CTOAs larger than 500 ms (not shown in the figures), without the expectation input the model does not show such a decrease in RTs.

These modeling results can be related to a behavioral study of Tipper and Kingstone [20], who performed a manipulation that, according to their interpretation, led participants to rely or not rely on the temporal predictability of cues. The condition with reliance on the temporal predictability replicated the common behavioral characteristics of IOR. In the condition without reliance on the temporal predictability IOR was still observed, but the RTs did not decrease with increasing CTOAs. These observations are consistent with the above-mentioned modeling results. Tipper and Kingstone, however, also reported a decrease in the magnitude of IOR in the condition without reliance on temporal predictability. A comparison of our Figures 3 and 6 shows that this decrease in not consistent with our results.

## Discussion

The activity of visuomotor neurons in the intermediate layer of the superior colliculus is closely related to IOR effects observed with behavioral measures [9] and the activity of these neurons has successfully been modeled with dynamic fields [16]. This suggests that IOR can also be modeled with dynamic fields. Satel and colleagues [14] indeed presented a single-layer dynamic field model that explains several IOR-related effects. However, IOR arguably emerges from the interaction of processes with different time-scales: a facilitatory effect of the cue-induced activation that decays quicker than the inhibitory effect of the cue-induced sensory habituation. The interaction of processes with different time-scales invites the use of multi-layer models.

We continued the direction taken by Satel and colleagues [14] and developed a multi-layer dynamic field model for IOR. Our model includes a dynamic account of sensory habituation. Simulations showed that (a) our model is consistent with behavioral IOR data [1], (b) the activation of visuomotor neurons in our model is qualitatively similar to the activity of visuomotor neurons in the intermediate layer of the superior colliculus of monkeys performing IOR tasks [9], (c) the sensory habituation shown by our model is qualitatively similar to the depression of the activity of neurons in the superficial layer of the superior colliculus of monkeys [11], and (d) our model is consistent with behavioral results regarding temporal expectancy effects [20].

With our model we aim to contribute to a dynamical-systems description of behavior as a multi-scale tuning to the environment [21], [22]. The shortest time-scale in our model is the one of the sensory field (τ*_S_* = .048 s^−1^), meaning that the sensory field has the highest temporal resolution. One order of magnitude above sensation, the decision field has a lower temporal resolution (τ*_D_* = .328 s^−1^), but it includes lateral interactions (Equations 6 and 7) that improve the spatial resolution. The decision layer integrates diverse sources of information so as to reach a unique decision. Our model includes a habituation that is one order of magnitude slower (τ*_D_* = 1.62 s^−1^) than the decision processes. The habituation layer is reciprocally coupled to the sensory layer (Equations 3 and 5). The habituation is crucial to the modeling of IOR. Even so, rather than being generated and localized at one of the layers, behavioral effects emerge from interactions among layers.

A tuning of the organism to the environment that occurs at yet longer scales can be referred to as learning. This process may be based on many perception-action cycles [23] and its effects may last for many cycles as well (cf. [24]). Our model does not implement processes at this longer time-scale. Relatedly, rather than a dynamic modeling of the expectation effect, our model assumes an expectation effect (see Equation 8 and the text above that equation). In the simulations the expectation effect is either included (in most cases) or not included (in the simulations concerning the results of [20]). To achieve a more encompassing model one could include an additional field, referred to as expectation field, whose activity builds up over trials at locations at which targets are presented, and with projections to the decision field (in analogy to the memory field discussed in [25]). Including processes with the longer time-scale of expectation may be needed to model empirically observed previous trial and learning effects [26]–[28].

To summarize, we developed a model that implements a multi-scale dynamic field explanation for IOR. Processes with different time-scales continuously and concurrently tune behavior to the environment. The model uses a single language, the language of dynamical systems, to describe informational, physiological, and behavioral quantities. The model combines these heterogeneous mechanisms into a single system composed of coupled state variables, extended over a common spatial metric and evolving in a common temporal dimension. Within such a framework, the behaviorally observed rates of the different phenomena are expressed as the time-scale separation of the dynamics of the different state variables.
